# Primary care engagement in health system change: a scoping review of common barriers and effective strategies

**DOI:** 10.1186/s12875-023-02117-2

**Published:** 2023-08-08

**Authors:** Michael Sergio Taglione, Judith Belle Brown

**Affiliations:** 1 Department of Family and Community Medicine, University of Toronto, 500 University Avenue, Toronto, ON M5G 1V7 Canada; 2 Institute of Health Policy, Management and Evaluation, University of Toronto, 155 College Street, Toronto, ON M5T 3M6 Canada; 3 Department of Family Medicine, Western University, 1465 Richmond Street, London, ON N6G 2M1 Canada

**Keywords:** Primary health care, Family medicine, Stakeholder engagement, Health system change, Health systems research, Scoping review

## Abstract

**Background:**

The complexity of health systems necessitates coordination between a multitude of stakeholders to enact meaningful change. Primary care physicians are a crucial partner to engage, as their investment and participation are critical to the success of any system-level initiative. The aim of this scoping review is to identify common barriers and effective strategies when engaging primary care physicians in designing and implementing health system change.

**Methods:**

A scoping review was performed. A literature search was performed in March 2020 using five databases. 668 unique articles were identified and underwent a title and abstract review. 23 articles met criteria for full text review and 10 met final inclusion criteria. A backward citation analysis identified two articles. 12 articles underwent data extraction and thematic analysis.

**Results:**

Several barriers to engagement were identified including a lack of trust between primary care physicians and decision-makers, strong professional physician identity, clinically irrelevant and complex proposals, and a lack of capacity and supports. Described strategies to overcome these barriers included building trust and relationships, contextual engagement strategies, working with physician leadership, enabling open and intentional communication channels, designing clinically relevant and straightforward initiatives, and considering financial incentives.

**Conclusions:**

Barriers to primary care engagement should be addressed with contextually designed strategies and a focus on relationship building, collaborative efforts, and implementing relevant and feasible initiatives. Further research should explore how to best develop relationships with primary care, working with collective voices of primary care physicians, and to better understanding the impact of financial incentives on engagement.

**Supplementary Information:**

The online version contains supplementary material available at 10.1186/s12875-023-02117-2.

## Background

Health systems are designed to provide comprehensive medical care for a defined population. These systems are constantly changing in pursuit of the Quadruple Aim, focused on achieving better outcomes, improved patient experience, lower healthcare costs, and improved clinical experience [1, 2]. Health systems are composed of numerous stakeholders including patients, healthcare providers, governments, hospitals, and private industry. Engaging these stakeholders is essential when implementing change.

Primary care is the backbone of well-functioning and comprehensive health systems focused on improving health outcomes and health equity [3, 4]. According to the World Health Organization, primary care has three core tenets: meeting people’s health needs through comprehensive care throughout the life course, systematically addressing the broader determinants of health, and empowering individuals, families, and communities to optimize their health [5]. Given the breadth of services provided, improving primary care can have significant impacts on individual and population health that include improved vaccination and cancer screening rates, lower total health costs, reductions in health equity disparities, and lower mortality rates [6–8].

In Canada, health system performance is measured among eight different categories: acceptability, accessibility, appropriateness, competence, continuity, effectiveness, efficiency, and safety [9]. Changes to the health system and primary care should aim to improve one or more of these factors. Despite efforts by governments and decision-makers around the world attempting to reform primary care, engaging primary care physicians to enable change aimed at improving these factors has proven difficult. As the service providers of primary care, physicians have significant influence in determining the success or failure of any reforms. Poor engagement can derail even the most well-intentioned and comprehensive plans for health system change.

Given the importance of primary care physicians, decision-makers need to be intentional in how they engage this group of stakeholders. However, engaging primary care physicians is poorly understood and inconsistently executed by governments and decision-makers. The purpose of this scoping review is to identify common barriers and effective strategies to consider when engaging primary care physicians in designing and implementing health system change.

## Methods

Scoping reviews are “exploratory projects that systematically map the literature available on a topic, identifying key concepts, theories, sources of evidence and gaps in the research” and “are often preliminary to full syntheses, undertaken when feasibility is a concern - either because the potentially relevant literature is thought to be especially vast and diverse or there is a suspicion that not enough literature exists” [10]. The methodological framework of Arksey and O’Malley and Levac et al. was used to guide the facilitation of the scoping review [11, 12]. The framework consists of: identifying the research question; identifying relevant studies; study selection; charting data; collating, summarizing, and reporting results; and stakeholder consultations.[11, 12]. Stakeholder consultations were facilitated concurrently with this review and those results will be published in the future. A protocol for this review was not published.

### Identifying the research question

The review’s objective was to identify common barriers and effective strategies when engaging primary care physicians in designing and implementing health system change.

### Identifying relevant studies

An initial search was performed in March 2020 on the following databases: PubMed, Cochrane Library, CINAHL, SCOPUS, and EMBASE. No date restrictions were applied to the search. Search terms included keywords related to family medicine, health system change, and engagement. Indexed subject headings were used in database searches when available. A research librarian from Western University was consulted to assist with search strategy development and execution. Search queries for each database are listed in Appendix 1.

### Study selection

**Fig. 1:**
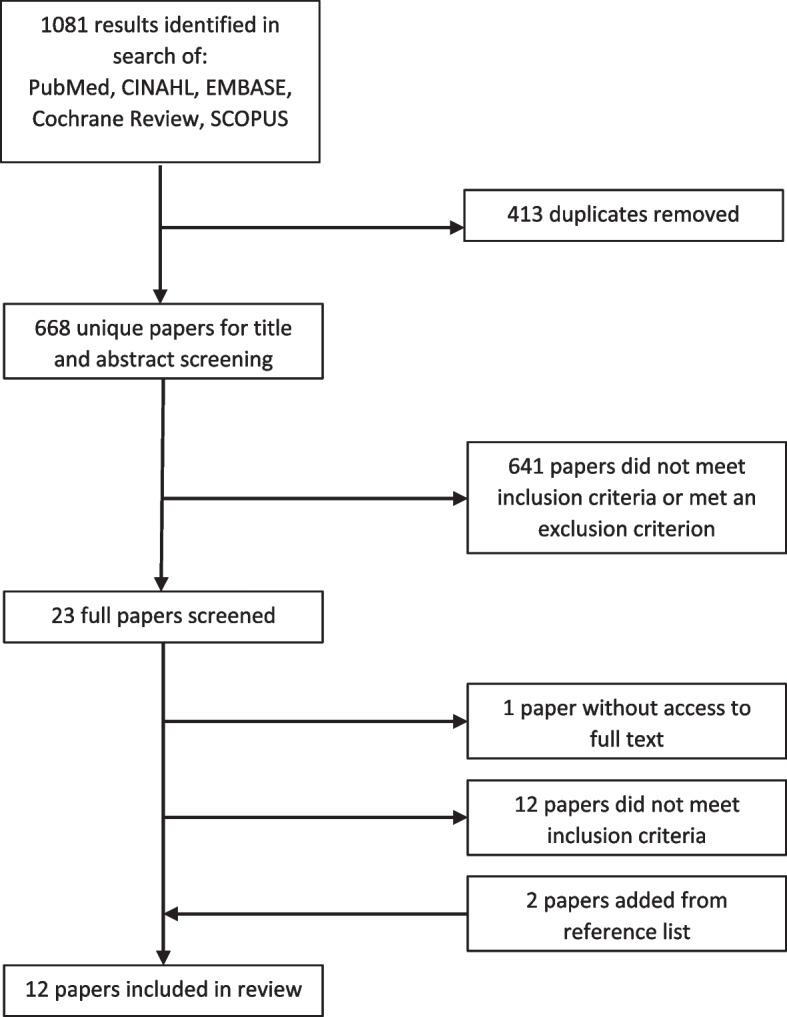
Article identification and selection flow diagram

1081 results were identified in the databases search (Fig. 1). A manual review of duplicates was performed by one author (MST) and 413 duplicates were removed. The remaining 668 unique articles underwent a title and abstract review to identify articles for full text review. The inclusion criteria of the title and abstract review were: (1) specific mention of primary care or family medicine, (2)engagement in health system change, and (3) articles written in English. The exclusion criteria were: (1)focus on primary care provided by other generalist groups (e.g. internal medicine, pediatrics) and or non-physician professions (e.g. nurse practitioners), and (2) focus on patient engagement.

Two reviewers performed the title and abstract review (MST and JBB). Both reviewers independently assessed 25 of the 668 articles for inclusion and exclusion criteria that were randomly selected using an online number generator. Reviewers discussed determinations to ensure consistency in their individual assessments. Reviewers then assessed the remaining articles’ titles and abstracts. Following the independent assessments, the reviewers resolved disagreements through discussion. A third party was not required to resolve disagreements. Of the 668 articles, 23 met criteria for full text review.

Full text review inclusion criteria were: (1) defines primary care as family medicine physicians or the equivalent in other countries, (2) focuses on at least one of three areas of primary care engagement in broad health system change (initiating engagement, maintaining engagement, or barriers and challenges in engaging primary care), and (3) articles written in English. The exclusion criteria were: (1) commentary articles, and (2) unavailable full text.

Two reviewers performed the full text review (MST and JBB). Both reviewers independently assessed 3 of the 23 articles for inclusion and exclusion criteria that were randomly selected using an online number generator. Reviewers discussed determinations to ensure consistency in their individual assessments. Reviewers then assessed the remaining articles’ titles and abstracts. Following the independent assessment, the reviewers resolved disagreements through discussion. A third party was not required to resolve disagreements. 10 articles met criteria for inclusion in the data extraction and analysis.

A backward citation analysis was then performed to identify articles from the reference sections of the 10 initially identified articles. Each reviewer (MST and JBB) assessed the titles of each reference. 321 titles were reviewed and 16 were identified by at least one reviewer for further evaluation of their abstracts. Five of the 16 articles met criteria for a full text review. Two articles met criteria for inclusion in the data extraction and analysis. The two articles underwent a repeated backward citation analysis. No relevant articles were identified from a total of 99 cited references.

### Charting data

12 articles met criteria and underwent data extraction and categorization. Categories included: Authors, Title, Year of Publication, Journal, Country of Origin, Type of Study, Primary Care Definition, Barriers to Engagement, Engagement Initiation Strategies, Engagement Maintenance Strategies, and Overarching Lessons Learned. Five of the 12 articles were randomly selected, and data extraction was performed by two reviewers (MST and JBB). After completion by both reviewers, a meeting was held to ensure consistency in their individual assessments. Data extraction of the remaining 7 articles was then performed by MST. The charted data for those 7 articles was reviewed by JBB prior to continuing to thematic analysis.

### Collating, summarizing and reporting the results

Qualitative thematic analysis was performed for the 12 included articles to group similar examples of barriers to engagement and strategies for effective engagement based on the information extracted in the Charting Data step. The two reviewers (MST and JBB) reviewed the charted data independently to establish common themes that addressed either barriers to engagement or strategies for effective engagement. The reviewers then met to discuss their independent analyses of the extracted data and discuss the overarching common barriers and strategies to effective engagement, which are described throughout the results section. A quantitative subgroup analysis of articles using quantitative or mixed methods was not performed.

## Results

### Study characteristics

Of the 12 articles included, six were conducted in Canada, three in the United States, two in the United Kingdom, and one in Australia (Table 1) [13–24]. Articles were published between 2014 and 2019. Nine of the 12 articles used a qualitative methodology, two used a quantitative methodology, and one used a mixed methods design.

**Table 1:** Selected articles that underwent data extraction

First author	Title	Year	Country	Participants (Total)	Aim of study	Method of data collection	Study conclusions quoted from abstracts
Abou Elnour, Amr	General practices’ perspectives on Medicare Locals’ performance are critical lessons for the success of Primary Health Networks.	2015	Australia	General practitioners (19), practice managers (18), practices nurses (15), community pharmacist (1)	To gather front-line staff’s perspectives on Medicare Locals and identify any lessons applicable to Primary Health Networks.	Individual semi-structured interviews	Those MLs that did well continued in an expanded way the work DGP were doing beforehand and made a seamless transition. PHNs will need to build on the strengths of previous PHOs, and create locality structures and processes that maximise the potential for clinical engagement. They will actively guide the dialogue between related microsystems: to achieve this they will have to be clinically led, change management organisations.
Ashman, Ian	Engaging with clinical commissioning: the attitudes of general practitioners in East Lancashire	2014	United Kingdom (England)	General practitioners (85)	To assess levels of engagement with clinical commissioning using a Clinical Commissioning Engagement Scale.	Standardized questionnaire	The findings highlight the potential challenges for CCGs in engaging GPs and in particular responding to perceived problems of capability and capacity. Further research is required to shed light on whether East Lancashire is typical of other CCGs.
Hanlon, Neil	Creating Partnerships to Achieve Health Care Reform: Moving Beyond a Politics of Scale?	2019	Canada	Community actors {managers, frontline providers, general practitioners, municipal leaders, community-based organizations} (65), executives (71)	To illuminate the ways in which competing logics of health care are expressed in and through a rhetoric of scale.	Individual semi-structured interviews	We examine points of tension between providers and administrators engaged in the reform process and show how these are often expressed discursively as a binary opposition involving central and local interests. We offer a critical examination of this politics of scale and seek to unpack claims of hierarchy and power as a means to offer insight into health care reform processes more generally.
Kreindler, Sara A.	The rules of engagement: physician engagement strategies in intergroup contexts	2014	USA	Organizational managers (58), primary care and specialty care physicians (51)	To utilize the social identity approach as a framework for examining how four disparate organizations engaged physicians in change.	Individual and group semi-structured interviews, observation of meetings and engagement events, review of documents	Beyond a universal focus on relationship-building, sites differed radically in their preferred strategies. Each emphasised or downplayed professional and/or organisational identity as befit the existing level of inter-group closeness between physicians and managers: an independent practice association sought to enhance members’ identity as independent physicians; a hospital, engaging community physicians suspicious of integration, stressed collaboration among separate, equal partners; a developing integrated-delivery system promoted alignment among diverse groups by balancing “systemness” with subgroup uniqueness; a medical group established a strong common identity among employed physicians, but practised pragmatic co-operation with its affiliates.
Kreindler, Sara A.	Primary care reform in Manitoba, Canada, 2011–15: Balancing accountability and acceptability	2019	Canada	Provincial and regional decision-makers (35) and primary care physicians (60)	To examine why the balance between accountability and acceptability remained elusive during a period of primary care reform in Manitoba, Canada from 2011–2015.	Individual and group semi-structured interviews, observation of meetings and engagement events, review of documents	Clearly delimited initiatives that directly promoted a specific observable behaviour (accountability) through financial or non-financial support (acceptability) were most successfully implemented. System-wide initiatives with complicated designs (notably a primary care network model that established formal partnership among clinics and regional health authorities) encountered greater difficulties in recruiting and sustaining physician participation. Although such initiatives offered physicians considerable decision-making latitude (acceptability), many physicians questioned the meaningfulness of opportunities for voice within a predetermined structure (accountability). Moreover, policymakers struggled to enhance the acceptability of such initiatives without sacrificing strong accountability mechanisms. Policymakers must carefully consider how acceptability and accountability elements may interact, and design them in such a way as to minimize the risk of mutual interference.
Kreindler, Sara A.	Pushing for partnership: physician engagement and resistance in primary care renewal.	2019	Canada	Family physicians (31), decision-makers (33)	To examine the difficulty faced by healthcare policymakers and managers in engaging family physicians in new models of primary care through a social identity lens.	Individual and group semi-structured interviews, observation of meetings and engagement events, review of documents	Recognizing that the existing physician–system relationship was generally distant, decision-makers invested effort in relationship-building. However, decision-makers’ rhetoric, as well as the design of their flagship initiative, evinced an attempt to proceed directly from interpersonal relationship-building to the establishment of formal intergroup partnership, with no intervening phase of supporting physicians’ group identity and empowering them to assume equal partnership. The invitation to partnership did not resonate with most physicians: many viewed it as an inauthentic offer from an out-group (“bureaucrats”) with discordant values; others interpreted partnership as a mere transactional exchange. Such perceptions posed barriers to physician participation in renewal activities.
McDermott, Imelda	Achieving integrated care through commissioning of primary care services in the English NHS: A qualitative analysis	2019	United Kingdom (England)	Policymakers (6), Clinical Commissioning Groups (CCGs) general practitioners and managers (42)	To analyse how CCGs have responded to new responsibilities and to identify challenges and factors that facilitated or inhibited achievement of integrated care systems.	Telephone surveys, interviews, observation of meetings	There is a disconnect between locally based primary care and the wider system. One of the major challenges we identified is the lack of knowledge and expertise in the field of primary care at STP level. While primary care commissioning by CCGs seems to be supporting local collaborations between practices, there is some way to go before this is translated into broader integration initiatives across wider footprints.
Pariser, Pauline	Improving System Integration: The Art and Science of Engaging Small Community Practices in Health System Innovation.	2016	Canada	Primary care providers/PCP (30)	To examine the perceived importance of various engagement strategies on initial PCP interest and on subsequent PCP participation in the project.	Standardized questionnaire	Project team acknowledgement that primary care is challenging and new access to patient resources were the most important factors in generating initial interest in SCOPE. The opportunity to improve patient care via integration with other providers was most important in their commitment to participate, and a positive experience with project personnel was most important in their continued engagement. Our experience suggests that such providers respond well to personalized, repeated, and targeted engagement strategies.
Pratt, Rebekah	Identifying Barriers to Collaboration Between Primary Care and Public Health: Experiences at the Local Level	2018	USA	Public health practitioners and administrators (20), primary care organization practitioners and administrators (20)	To identify barriers to collaboration between primary care and public health at the local level in 4 states.	Individual semi-structured interviews	Some barriers to collaboration (e.g., changes to health care billing, demands on provider time) require systems change to overcome, whereas others (e.g., a lack of shared priorities and mutual awareness) could be addressed through educational approaches, without adding resources or making a systemic change. Overcoming these common barriers may lead to more effective collaboration.
Reay, Trish	Getting leopards to change their spots: Co-creating a new professional role identity	2017	Canada	Family physicians (63), other health professionals (26), and managers (73)	To analyze efforts over time to change physicians’ collective professional role identity.	Individual semi-structured interviews, archival government/ AMA/PCN document review	We found that the change in physician professional role identity required significant identity work by a group of actors, but particularly by the managers who had been charged with leading the reform initiative. We contribute to the professional role identity and institutional literatures by showing how others can engage in social interactions with professionals to facilitate the reinterpretation and rearranging of institutional logics that guide collective professional role identity.
Skillman, Megan	Physician Engagement Strategies in Care Coordination: Findings from the Centers for Medicare & Medicaid Services’ Health Care Innovation Awards Program.	2017	USA	Primary care and specialty care physicians (95), program staff/leadership and program partners (577)	To identify roles physicians assumed as part of new health care delivery models and related strategies that facilitated physician engagement across 21 Health Care Innovation Award programs.	Individual and group semi-structured interviews, program observation	We describe engagement strategies derived from a diverse range of programs. Successful programs considered physicians’ values and engagement as components of process and policy, rather than viewing them as exogenous factors affecting innovation adoption. These types of approaches enabled programs to accelerate acceptance of innovations within organizations.
Snadden, David	Engaging primary care physicians in system change - An interpretive qualitative study in a remote and rural health region in Northern British Columbia, Canada	2019	Canada	Family physicians (10), non physician division leads (3), primary care coordinators (18), regional health authority leaders (3)	To describe how physicians were engaged in primary healthcare system change in a remote and rural Canadian health authority.	Individual semi-structured interviews	Physician engagement was recognised as a priority by Northern Health in its efforts to create system change. This was facilitated by the creation of Divisions of Family Practice that provided a structure for dialogue and facilitated a common voice for physicians. Divisions helped to build trust between various groups through allowing constructive conversations to surface and deal with tensions. Local context mattered. Flexibility in working from local priorities was a critical part of developing relationships that facilitated the design and implementation of system reform.

Articles were assessed for findings that described barriers to engagement and strategies for the initiation and maintenance of engagement. Eleven of the twelve articles discussed barriers and ten of the twelve articles discussed initiation and maintenance strategies. Quotations extracted from the articles describing the barriers to engagement and strategies for effective engagement can be found in Tables 2 and 3, respectively.

**Table 2:** Extracted quotations illustrating barriers to change

Barrier to change	Extracted quotations
Lack of trust and poor relationships between primary care and decision-makers	“Participants pointed to concerns regarding who was having conversations with whom, and the historical mistrust between professionals and health authorities.” [13]. “Regional actors had kept the frontline out of the early stages of network consultations [and] many community actors still considered the omission of frontline personnel from planning and partnering efforts as a clear indication that the initiative was, ultimately, a “top-down” undertaking.” [14]. “One of the major challenges to integrate care vertically or ‘knitting’ the locally-based primary care plan with regional plans… is the lack of knowledge and expertise in the field of primary care at the [sustainability and transformation partnerships] level.” [18].
Strong professional physician identity	“Directives from ‘above’ to make changes in these daily routines are expected to face resentment and resistance. When considered in connection with ideas of professional autonomy and the sanctity of clinical judgment, frontline personnel and their cultures of practice present potentially substantial barriers to the implementation of these reforms.” [14]. “I think there’s a cohort of people who see it as an opportunity to shape the future and then there’s a cohort of people who think, you know, it’s concerning about the future of general practice.” [18]. “Physicians said that they listened carefully to the [Alberta Medical Association’s] concerns, and they were more leery of full participation in the [Primary Care Network] as a result.” [19].
Clinically irrelevant and complex proposals for change	“Identified barriers to engagement of primary care physicians include limited time and resources, lack of information technology and staff support, and the perception that proposed interventions are either irrelevant or impractical to day-today practice.” [20]. “The slow, bureaucratic nature of decision-making bred frustration and alienation; and the loose, conceptual definition of [My Health Teams] was a source of confusion and even some suspicion.” [21]
Lack of capacity and supports	“Participants cited limited time, capacity, or resources to develop new work or new partnerships in the face of struggling to just “keep the lights on” for current services.” [15].

**Table 3:** Extracted quotations illustrating strategies for effective engagement

Strategy for effective engagement	Extracted quotations
Building trust and collaborative relationships	“The maintenance of trust could not be assumed… Once trust was developed it was just as important to find some early wins to show progress was being made.” [13]. “Prioritizing policies that encourage aligned planning processes for both primary care and public health could bring partnerships together to explore and identify shared priorities for limited resources. Undertaking shared strategic planning may help partnerships identify and prioritize barriers to address collaboratively” [15].
Targeted engagement strategies as one size does not fit all	“A very personal and iterative approach [was used] to engaging [primary care physicians]. This approach required a high level of oversight by the team and [primary care lead], which is resource intensive and may be challenging to scale to other sites.” [20].
Physician leadership and collective voice	“Leveraging physician champions and establishing innovation-values fit between programs and physicians were critical parts of engagement. In addition to generating a positive innovation climate, these approaches informed innovation policies and procedures as well as how programs tried to prove their value to physicians, often through emphasizing increased workflow efficiency and minimal time investment.” [23]. “Working with structures that were designed to give physicians a collective voice helped build relationships, find common ground, encourage dialogue and enhance continuity.” [13].
Open and intentional communication strategies	“The tensions identified in the interviews were often recalled as hidden and unacknowledged in the interactions between partners… Honest conversations and structures for communication were necessary. Through conscious dialogue, they could surface and work through tensions that developed when changes were made to how services were designed and delivered. These efforts have not been easy or straightforward. They have taken a long period of time, as foundations of commonly agreed-upon and deliberately purposeful actions have required an understanding of others’ contexts.” [13]. “Whereas an earlier narrative included the term ‘rolling-out reform,’ senior administrators later spoke of ‘facilitating reform.’ There was also an effort to brand their new approach as the ‘Northern Health way’.” [14].
Clinically relevant initiatives and straightforward initiatives	“Approaches like this allowed NH and physicians to develop working relationships focused on improving care for the people they served, which allowed tensions to be identified, managed and worked through. Actions were focused on what could be done together to improve patient care, such as the creation of an unattached patient clinic, the development of a family practice clinical teaching unit and actively helping people learn about others’ working contexts.” [13]. “The initiatives most likely to meet their objectives were those in which acceptability elements directly facilitated physicians’ achievement of an outcome for which they were accountable. Such direct, meaningful relatedness between acceptability and accountability was exemplified by initiatives that provided support for a specific, easily observable behaviour, such as electronic medical record adoption or patient attachment.” [21].
Financial incentives	“[Primary care networks] were set up to be attractive to physicians – proposals suggested that family physicians could improve work/life balance, improve quality of care for patients, and receive small financial incentives for engaging in planning processes. Physicians were reimbursed for time spent at meetings, program development and other planning activities, that were otherwise not funded. In addition, money was available to hire a wide range of health professionals.” [19].

### Barriers to engagement

Four common barriers to engagement of primary care physicians by governments and decision-makers were identified among the articles: (1) lack of trust and poor relationships between primary care and decision-makers; (2) strong professional physician identity; (3) clinically irrelevant and complex proposals for change; and (4) lack of capacity and supports.

#### Lack of trust and poor relationships between primary care and decision-makers

Distrust in administration and governments often preceded change initiatives and was a significant hurdle to overcome when initiating the engagement process [13]. Exclusion of primary care in early discussions further exacerbated distrust and poor communication hindered collaborative efforts [14–17]. In some instances, physicians described differences in core values between physician and non-physician groups [17]. Others described a lack of primary care knowledge among decision-makers, leading to challenges in initiatives aimed at integrating care [18].

#### Strong professional physician identity

Professional autonomy and a strong physician identity were significant barriers. Primary care physicians desired control of clinical decision making and often viewed themselves as functioning independently of the larger health system [13, 14]. In some instances, change efforts created a spectrum of differing physician opinions [18]. Established physician groups that strengthened the physician voice could make change efforts quite difficult if the group were not in favour of the proposed change [19].

#### Clinically irrelevant and complex proposals for change

Primary care physicians were reluctant to engage if they felt interventions were unlikely to be clinically relevant or difficult to implement [20]. Unclear interventions and fears of increased administrative work without clinical improvement led to confusion and frustration that hampered efforts to further discussions [21].

#### Lack of capacity and supports

Lack of organizational and administrative supports for primary care physicians were deterrents to engagement [22]. Limited time and capacity further prevented engagement [15].

### Strategies for effective engagement

Six strategies for effective engagement of primary care physicians by governments and decision-makers were identified among the articles: (1) building trust and collaborative relationships; (2) targeted engagement strategies as one size does not fit all; (3) physician leadership and collective voice; (4) open and intentional communication strategies; (5) clinically relevant initiatives and straightforward initiatives; and (6) financial incentives.

#### Building trust and collaborative relationships

Many studies highlighted trust as a key contributor to relationship building and cited both formal and informal efforts to develop these relationships with clinicians [13, 21]. For example, local administrators and primary care coordinators involved in a rural primary care setting were able act as translators between regional administrators and physicians because of predeveloped relationships, enabling dialogue that resulted in co-design of new programs and delivery of services [13].

Efforts to build trust were time-intensive and in some instances took years to develop [14]. Once relationships had been established, ongoing efforts were required to maintain them [13]. Fostering these relationships allowed for interdisciplinary partnerships to partake in shared strategic planning efforts [15].

#### Targeted engagement strategies as one size does not fit all

Many studies described generic engagement strategies like town-halls and newsletters. However, the most effective strategies were those created to engage a specific audience [24]. These specific strategies were effective, but also resource intensive [20].

#### Physician leadership and collective voice

Physician champions were a well-established role in engaging a larger primary care audience [23]. Beyond the leadership of individual physicians, an organized and credible collective physician voice sometimes provided a clear method for reaching a larger group to engage [13].

#### Open and intentional communication strategies

An open, non-judgemental communication channel was important in initiating engagement with physicians, especially for new or strained relationships [13]. Messaging mattered, as seen in instances where unclear language confused primary care physicians resulting in unproductive engagement efforts [17]. Intentional phrasing and communication strategies focused on developing a collaborative relationship with primary care physicians were helpful in cultivating ongoing engagement efforts [14].

#### Clinically relevant and straightforward initiatives

Administrators were able to improve engagement efforts by focusing initiatives on areas with clear efforts to improve patient care [13]. Physicians were more likely to buy in to initiatives if they were uncomplicated and tied to an observable measurement [21].

#### Financial incentives

Only one study had explicitly described providing funding to incentivize engagement. It was described as an effective strategy in engagement efforts [19]. However, few studies explored the role financial incentives had in promoting engagement. Some studies referenced articles that suggested financial incentives did not change quality of care [20, 23].

## Discussion

Based on this review, there does not seem to be established best practices for engaging primary care, but common barriers are seen throughout the studies. Different combinations of strategies to overcome these barriers were implemented, though there was not one specific intervention that determined whether engagement efforts would succeed. Instead, targeted engagement efforts unique to the context and focused on mitigating commonly seen barriers seemed to provide the most benefit.

The engagement strategies described in the twelve studies, both successful and unsuccessful, provided the following lessons to consider when engaging primary care physicians.

### Focus efforts on relationship building

A lack of trust between primary care and decision-makers, and the difficult relationships that resulted, was the most cited barrier to engaging primary care. Successful decision-makers prioritized establishing trust and repairing strained relationships as needed. Many differing tactics were used, but the most effective were those tailored to the specific group of local primary care physicians. Generic strategies like townhalls and newsletters improved communication and transparency, but true relationship building required significant time and intentional efforts. Targeted strategies, however, can be resource intensive and unfeasible in many circumstances, particularly with a provincial or national scope of change.

Clear communication was crucial in building strong, meaningful relationships. Intentional language that emphasized joint efforts and improving meaningful patient, provider, and health system outcomes is important when engaging primary care physicians.

### Collaborate with primary care physician champions and primary care physician groups

Primary care physician champions were effective in building on pre-existing relationships to engage their local primary care community. They served local physicians as a direct line to decision-makers while simultaneously facilitating communication of initiative details to physicians for decision-makers.

Primary care physician groups composed of trusted local primary care leaders were able to speak as a collective and credible physician voice that engaged more physicians and had more impact in adjusting proposed initiatives. These groups were often able to unite local physicians, although opinions did not always align with those of administration. However, connecting with these groups was more streamlined for administrator engagement efforts.

Although physician champions and primary care physician groups cannot always engage with some dissenting physicians, both represent a credible, trusted physician voice and can be crucial partners to engage early when implementing health system change.

### Ensure change Ideas are clinically relevant and feasible

Initiatives that were not clinically relevant quickly lost the support and engagement of primary care physicians. Low physician capacity and poor supports also hindered the uptake of change initiatives. Complicated initiatives also faced difficulty in achieving buy in compared to more straightforward solutions. Changemakers who focus on creating clinically relevant initiatives that improved primary care physician workload will be more successful in engaging physicians.

### Consider use of financial incentives

Financial incentives have not been extensively studied in how they impact primary care engagement. A few studies extrapolated research that financial incentives do not improve quality of care and expressed financial incentives do not improve engagement. One study used financial incentives by paying physicians for time spent attending meetings and participation in program development [19]. The incentives were described to be effective at achieving engagement in a single study, but further investigation into the effectiveness and sustainability of this strategy is needed.

### Limitations

Limitations in this scoping review include the timing of the review and the sources of research. The literature search was performed March 2020 and thus any subsequent publications discussing primary care engagement in system level change, specifically around primary care engagement in response to the COVID-19 pandemic, would not have been included in this scoping review. Grey literature reports were not included in the analyses and may not have identified themes exclusively described outside academic literature. Although a backward citation analysis was performed, a forward citation analysis was not and may have identified additional articles that met study criteria. Data extraction of 7 articles was performed by one reviewer and results were reviewed by the second rather independently assessed.

## Conclusion

A scoping review was performed to identify common barriers and effective strategies to consider when engaging primary care physicians in designing and implementing health system change. Twelve articles were identified through a literature search and backward citation analysis. Commonly identified barriers included poor trust and relationships, strong professional physician identity, clinically irrelevant and complex initiatives, and a lack of capacity and supports. Some of these were addressed by commonly used strategies including efforts focused on relationship building, collaborating with primary care physician champions and primary care physician groups, and ensuring initiatives are clinically relevant and straightforward to implement. Although there are no published best practices available for engaging primary care, focused efforts that work to mitigate common barriers should see better engagement results.

Current work is underway by JBB exploring the role primary care leaders in health system change and how to define and measure the meaningful involvement of primary care [25]. Further research is required to better understand how to best build trust and develop relationships with primary care physicians and primary care groups. Additionally, the use of financial incentives was suggested as a strategy by one study, but further research is required to understand the impacts of providing financial incentives in initiating and maintaining engagement.

### Supplementary Information

**Additional file 1: Appendix 1. **Literature search strategy.

## Data Availability

All data generated or analysed during this study are included in this published article and its supplementary information files.

## Abbreviations

MST: Michael Sergio Taglione (author)
JBB: Judith Belle Brown (author)
