# Supplementary material for: Primary care engagement in health system change: a scoping review of common barriers and effective strategies
Source: BMC Prim Care. 2023 Aug 8;24:157. doi: 10.1186/s12875-023-02117-2 (PMC10408209; doi:10.1186/s12875-023-02117-2)
Supplement: Supplementary file 1 — Additional file 1: Appendix 1. Literature search strategy. [file 12875_2023_2117_MOESM1_ESM.docx]

**Appendix 1: Literature Search Strategy**

*PubMed search strategy performed March 23, 2020*

(((((((((((((((((((((“general practice”) OR “general practitioners”) OR “general practitioner”) OR “family physicians”) OR “family physician”) OR “family doctors”) OR “family doctor”) OR “family medicine”) OR “family practice”) OR “primary health care”) OR “primary care”) OR “primary healthcare”) OR “general practice”[MeSH Terms]) OR “general practitioners”[MeSH Terms]) OR “physicians, family”[MeSH Terms]) OR “family practice”[MeSH Terms]) OR “primary health care”[MeSH Terms]) OR "primary care provider") OR "primary care providers")) AND ((((((((((((((((“Healthcare reform”) OR “System reform”) OR “Systems reform”) OR “Healthcare change”) OR “Health care reform”) OR “System change”) OR “Systems change”) OR “Healthcare design”) OR “Health care design”) OR “System design”) OR “Systems design”) OR “Healthcare innovation”) OR “Health care innovation”) OR “System innovation”) OR “Systems innovation”) OR “Health care reform”[MeSH Terms])) AND (((((“Engagement”) OR “Codesign”) OR “Co-design”) OR “Partner”) OR “Partnership”)

*CINAHL search strategy performed March 23, 2020*

S1: “general practice” OR “general practitioners” OR “general practitioner” OR "family physicians” OR “family physician” OR “family doctors” OR “family doctor” OR “family medicine” OR “family practice” OR “primary health care” OR “primary care” OR “primary healthcare”

S2: MW “general practice” OR MW “general practitioners” OR MW “physicians, family” OR MW “family practice" OR MW “primary health care"

S3: S1 OR S2

S4: “Healthcare reform” OR “System reform” OR "Systems reform” OR “Healthcare change” OR “Health care reform” OR “System change” OR “Systems change” OR “Healthcare design” OR “Health care design” OR “System design” OR “Systems design” OR “Healthcare innovation”

S5: “Health care innovation” OR “System innovation” OR “Systems innovation” OR MW “Health care reform”

S6: S4 OR S5

S7: “Engagement” OR “Codesign” OR “Co-design” OR “Partner” OR “Partnership”

S8: S3 AND S6 AND S7

*SCOPUS search strategy performed March 23, 2020*

( TITLE-ABS-KEY-AUTH ( "general practice" OR "general practitioner*" OR "family physician*" OR "family doctor*" OR "family medicine" OR "family practice" OR "primary health care" OR "primary care" OR "primary healthcare" ) ) AND ( TITLE-ABS-KEY-AUTH ( "Healthcare reform" OR "System reform" OR "Systems reform" OR "Healthcare change" OR "Health care reform" OR "System change" OR "Systems change" OR "Healthcare design" OR "Health care design" OR "System design" OR "Systems design" OR "Healthcare innovation" OR "Health care innovation" OR "System innovation" OR "Systems innovation" ) ) AND ( TITLE-ABS-KEY-AUTH ( "Engagement" OR "Codesign" OR "Co-design" OR "Partner" OR "Partnership" ) )

*EMBASE search strategy performed March 23, 2020*

1. (general practice or general practitioners or general practitioner or family physicians or family physician or family doctors or family doctor or family medicine or family practice or primary health care or primary care or primary healthcare).af. or general practice.sh. or general practitioners.sh. or physicians, family.sh. or family practice.sh. or primary health care.sh.

2. (Healthcare reform or System reform or Systems reform or Healthcare change or Health care reform or System change or Systems change or Healthcare design or Health care design or System design or Systems design or Healthcare innovation or Health care innovation or System innovation or Systems innovation).af. or Health care reform.sh.

3. (Engagement or Codesign or Co-design or Partner or Partnership).af.

*Cochrane Library search strategy performed March 23, 2020*

(“general practice” OR “general practitioners” OR “general practitioner” OR “family physicians” OR “family physician” OR “family doctors” OR “family doctor” OR “family medicine” OR “family practice” OR “primary health care” OR “primary care” OR “primary healthcare”) in All Text AND (“Healthcare reform” OR “System reform” OR “Systems reform” OR “Healthcare change” OR “Health care reform” OR “System change” OR “Systems change” OR “Healthcare design” OR “Health care design” OR “System design” OR “Systems design” OR “Healthcare innovation” OR “Health care innovation” OR “System innovation” OR “Systems innovation”) in All Text AND (“Engagement” OR “Codesign” OR “Co-design” OR “Partner” OR “Partnership”) in All Text - (Word variations have been searched)
